# Cetuximab-Induced MET Activation Acts as a Novel Resistance Mechanism in Colon Cancer Cells

**DOI:** 10.3390/ijms15045838

**Published:** 2014-04-04

**Authors:** Na Song, Shizhou Liu, Jingdong Zhang, Jing Liu, Ling Xu, Yunpeng Liu, Xiujuan Qu

**Affiliations:** Department of Medical Oncology, the First Hospital of China Medical University, Shenyang 110001, China; E-Mails: songna_cmu@hotmail.com (N.S.); liu_shizhou@hotmail.com (S.L.); jd_zhang@hotmail.com (J.Z.); liujing_cmu@hotmail.com (J.L.); cmuxuling@hotmail.com (L.X.)

**Keywords:** cetuximab, MET, EGFR, SRC, colon cancer

## Abstract

Aberrant MET expression and hepatocyte growth factor (HGF) signaling are implicated in promoting resistance to targeted agents; however, the induced MET activation by epidermal growth factor receptor (EGFR) inhibitors mediating resistance to targeted therapy remains elusive. In this study, we identified that cetuximab-induced MET activation contributed to cetuximab resistance in Caco-2 colon cancer cells. MET inhibition or knockdown sensitized Caco-2 cells to cetuximab-mediated growth inhibition. Additionally, SRC activation promoted cetuximab resistance by interacting with MET. Pretreatment with SRC inhibitors abolished cetuximab-mediated MET activation and rendered Caco-2 cells sensitive to cetuximab. Notably, cetuximab induced MET/SRC/EGFR complex formation. MET inhibitor or SRC inhibitor suppressed phosphorylation of MET and SRC in the complex, and MET inhibitor singly led to disruption of complex formation. These results implicate alternative targeting of MET or SRC as rational strategies for reversing cetuximab resistance in colon cancer.

## 1. Introduction

New approaches targeting epidermal growth factor receptor (EGFR) have clearly improved the clinical benefits in several solid tumors, including colorectal cancer (CRC). Cetuximab (C225, Erbitux) is a chimeric mouse-human monoclonal antibody that specifically binds the extracellular domain of EGFR, subsequently blocking the downstream signaling of EGFR that influences cell proliferation, survival, apoptosis, migration and tumorigenesis. In clinical trials, cetuximab has exhibited promising antitumor activity as a monotherapy and in combination with chemotherapy as first-line treatment for metastatic CRC. Pooled analysis of the CRYSTAL and OPUS clinical trials showed better response rate, progression-free survival and overall survival when addition of cetuximab to chemotherapy in *KRAS* wide-type patients [1]. However, the therapeutic efficacy of cetuximab is ultimately limited by the emergence of mutations and other mechanisms that confer drug resistance.

*KRAS* mutations, which are seen in 35%–40% of CRCs, have emerged as the most important predictive biomarker in selecting patients who will benefit from cetuximab [2]. Recently, *NRAS* mutations have emerged as an indicator for EGFR-targeted agent [3]. In addition to *RAS* mutational status, some studies have demonstrated that oncogenic activation of effectors downstream of EGFR, such as mutant *BRAF*, *PIK3CA*, and *PTEN* inactivation, are associated with cetuximab resistance [4,5]. However, approximately 25% of CRC patients with wild-type *KRAS*, *BRAF*, *PIK3CA* and *PTEN* do not respond to cetuximab, and the resistance mechanism is still unknown. Besides gene mutation, multiple resistance mechanisms to cetuximab include overexpression of EGFR ligands and receptors, ubiquitylation, translocation of EGFR, EGFR variant III, modulation of EGFR by SRC family kinases, and transactivation of alternative pathways that bypass the EGFR pathway [6].

Increasing evidence indicates that MET, the tyrosine kinase receptor for hepatocyte growth factor (HGF), is frequently implicated in resistance to EGFR-targeted therapies, including EGFR tyrosine kinase inhibitors (TKIs) and EGFR antibodies [7–9]. A recent study has demonstrated that HGF-dependent MET activation contributes to cetuximab resistance in colon cancer [10]. Moreover, there exists ligand-independent MET activation caused by gene amplification, overexpression, mutation, autocrine stimulation, transactivation by other membrane proteins, or loss of negative regulators [11]. Sometimes, the induced activation of signaling pathway by targeted drug will drive resistance. In EGFR TKI erlotinib-resistant lung cancer cells and colon cancer cells, the induced insulin-like growth factor-I receptor activation is implicated in resistance to erlotinib [12,13]. However, whether the induced MET activation by EGFR inhibitors mediating resistance is less understood. An important intermediary connecting MET with EGFR is SRC non-receptor kinase [14]. In breast cancer cells, MET and SRC cooperate to compensate for the loss of EGFR TKI activity [15]. Furthermore, SRC activation is a common mechanism for resistance to HER2 and EGFR inhibitors [16,17].

In this study, we demonstrated that MET activation induced by cetuximab was involved in resistance to cetuximab in colon cancer cells. Additionally, we further confirmed that the interaction between MET and SRC and the formation of MET/SRC/EGFR complex contributed to constitutive MET activation, providing a rationale for combinatorial inhibition of EGFR and MET or EGFR and SRC in therapy targeting colon cancer.

## 2. Results

### 2.1. Cetuximab Induces MET Activation in Cetuximab-Insensitive Caco-2 Cells

**Figure 1.**
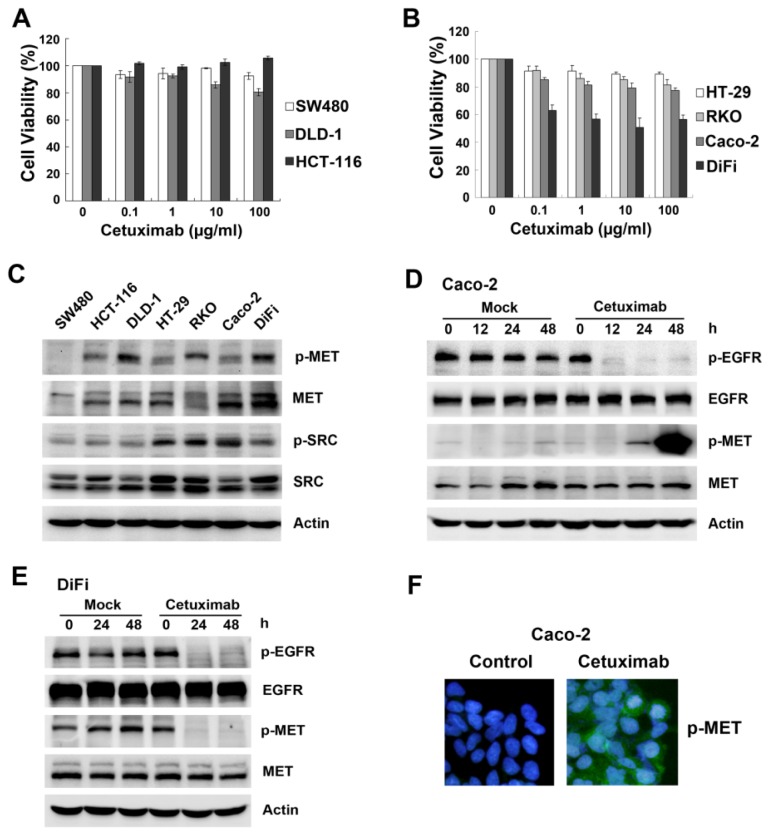
Cetuximab induces MET phosphorylation in cetuximab-insensitive Caco-2 cells but not in cetuximab-sensitive DiFi cells. (**A**,**B**) Three *KRAS* mutant colon cells (SW480, DLD-1 and HCT-116) and four *KRAS* wide-type colon cells (HT-29, RKO, Caco-2 and DiFi) were treated with increasing concentrations of cetuximab (0.1, 1, 10, 100 μg/mL) for 72 h after overnight 2% FBS starvation. Cell viability was determined by MTT assay; (**C**) Expression of MET, SRC and phosphorylation levels were examined by Western blotting in seven representative colon cells. Actin was shown as loading control for all Western blotting; (**D**,**E**) Caco-2 cells and DiFi cells were treated with 10 μg/mL cetuximab for the indicated times. Expression of MET, EGFR and phosphorylation levels were analysed by Western blotting. Caco-2 cells were treated with 10 μg/mL cetuximab for 48 h in 2% FBS medium; (**F**) Cyto-staining for MET phosphorylation on Tyr1234/1235 (green) and nuclei (blue) were detected by immunofluorescence.

Overexpression or activation of MET and SRC are reported to correlate with primary resistance to EGFR inhibitors in several solid tumors [18–21]. To investigate the mechanism of resistance to cetuximab in colon cancer cells, we first tested the effect of cetuximab on cell proliferation and basal MET and SRC protein expression and phosphorylation in seven colon cancer cell lines, including three mutant *KRAS* lines (SW480, HCT-116, DLD-1) and four wild-type *KRAS* lines (HT-29, RKO, Caco-2 and DiFi). MTT assays revealed varying anti-proliferative activity of cetuximab, which was cell line-dependent (cell viability of 10 μg/mL cetuximab at 72 h is shown in Supplementary Table S1). DiFi cells were sensitive to cetuximab, while all other cell lines tested were insensitive or resistant to cetuximab, even those that were wild-type for *KRAS* (Figure 1A,B). Next, the expression of phosphorylated and total MET and SRC was evaluated by Western blotting; the variable expression of these proteins did not correlate with cetuximab response in colon cancer cells (Figure 1C).

Next, changes in the MET pathway following cetuximab treatment were observed. Given that gene mutations (*KRAS*, *BRAF* and *PIK3CA*) influence cetuximab sensitivity, we chose two cell lines (Caco-2 and DiFi) that express wild-type *KRAS*, *BRAF* and *PIK3CA*, paralleling the molecular features of colon cancer patients who are most likely to respond to cetuximab (Supplementary Table S1). Phosphorylated EGFR was suppressed by cetuximab in both lines, as predicted. Notably, cetuximab-induced MET phosphorylation was elevated at 24 h and peaked at 48 h in cetuximab-insensitive Caco-2 cells (Figure 1D), whereas MET phosphorylation was inhibited in cetuximab-sensitive DiFi cells (Figure 1E). Furthermore, enhanced phospho-MET expression in the presence of cetuximab was confirmed by staining (Figure 1F). The different levels of MET activation due to cetuximab indicated that induced activation of the MET pathway was involved in the response to cetuximab.

### 2.2. Cetuximab-Induced MET Activation Contributes to Cetuximab Resistance

**Figure 2.**
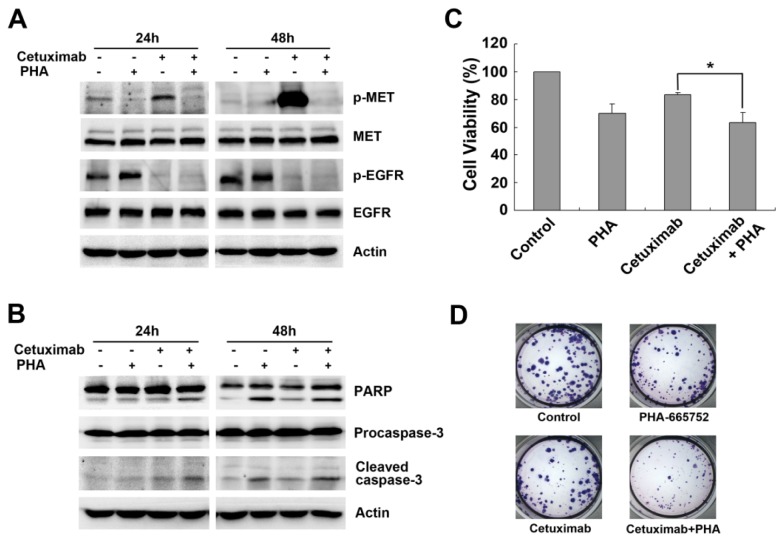
MET inhibitor PHA-665752 inhibits MET phosphorylation induced by cetuximab in Caco-2 cells. (**A**,**B**) Caco-2 cells were treated with cetuximab (10 μg/mL), PHA-665752 (0.4 μM) or both for 24 and 48 h in 2% FBS medium. Expression of MET, EGFR and phosphorylation levels, PARP and Caspase-3 were analyzed by Western blotting; (**C**) Caco-2 cells were treated with 10 μg/mL cetuximab or combination with 0.4 μM PHA-665752 for 48 h in 2% FBS medium, then followed by MTT assay. Results from three independent experiments are shown. Student’s *t*-test, * *p* < 0.05 for comparisons between cetuximab-treated and combined treatment; (**D**) Caco-2 cells were incubated with either 10 μg/mL cetuximab or 0.4 μM PHA-665752, or in combination treatment for 14 days, and the clones were counted.

To investigate the consequences of MET activation on cetuximab sensitivity, we treated Caco-2 cells with a selective MET inhibitor, PHA-665752. As expected, PHA-665752 treatment combined with cetuximab was sufficient to diminish cetuximab-induced MET phosphorylation in Caco-2 cells (Figure 2A). Moreover, PHA-665752 enhanced the apoptotic effect; combined treatment with cetuximab and PHA-665752 induced significant apoptosis as detected by PARP and Caspase-3 cleavage (Figure 2B). Therefore, combined inhibition of EGFR and MET effectively inhibited cell proliferation, as detected by MTT assay (63.12% ± 7.22% *vs.* 83.49% ± 1.46%, *p* < 0.05, Figure 2C), and colonies formed in the presence of the combined treatment were smaller and fewer in number (Figure 2D). These results confirmed that delayed MET activation resulted in cetuximab resistance by activating MET.

### 2.3. Downregulation of MET by siRNA Restores Cetuximab-Induced Cell Proliferation Inhibition

**Figure 3.**
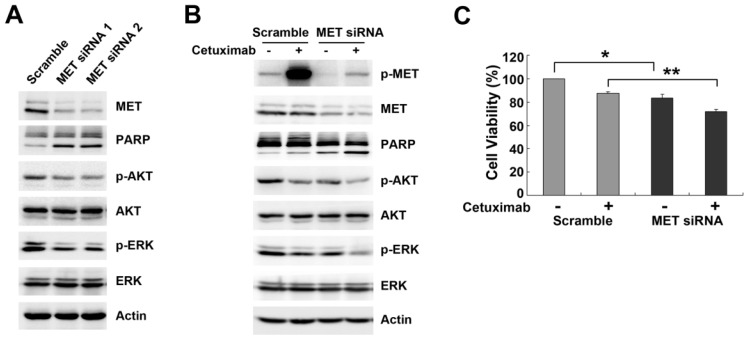
Knockdown of MET by siRNA in Caco-2 cells restores sensitivity to cetuximab. (**A**) Caco-2 cells were transiently transfected with two pairs of MET siRNA separately and Scramble Control siRNA for 48 h. Knockdown of MET and successive inhibition of downstream targets were examined by Western blotting; (**B**,**C**) Caco-2 cells were transfected with Scramble Control siRNA or MET siRNA followed by treatment with 10 μg/mL cetuximab for 48 h in 2% FBS medium. Cell lysates were subjected to Western blotting, and cell viability was assessed by MTT assay. The results from three independent experiments are shown. Student’s *t*-test, * *p* < 0.01, ** *p* < 0.001 for comparisons between two arms.

To further explore the role of MET in cetuximab resistance, we used MET siRNA to determine the effect of MET downregulation on cetuximab sensitivity in Caco-2 cells. Knockdown of MET abolished cetuximab-induced MET activation and resulted in enhanced apoptosis, as measured by PARP expression and inhibition of phospho-AKT and phospho-ERK levels (Figure 3A). Dual inhibition with MET siRNA and cetuximab effectively induced cleavage of PARP and augmented inhibited phosphorylation of AKT and ERK (Figure 3B). Furthermore, disruption of MET signaling led to effective inhibition of cell proliferation (83.5% ± 2.95% *vs.* 100%, *p* < 0.01, Figure 3C), and further treatment with cetuximab following MET downregulation resulted in further decrease in cell viability (71.97% ± 1.69% *vs.* 87.25% ± 1.4%, *p* < 0.001, Figure 3C), confirming that MET-promoted intracellular signaling drove resistance to cetuximab. Taken together, these data suggested MET activation provided an alternative pathway that bypassed EGFR blockade by cetuximab in Caco-2 cells.

### 2.4. SRC Mediates Cetuximab-Induced MET Activation

**Figure 4.**
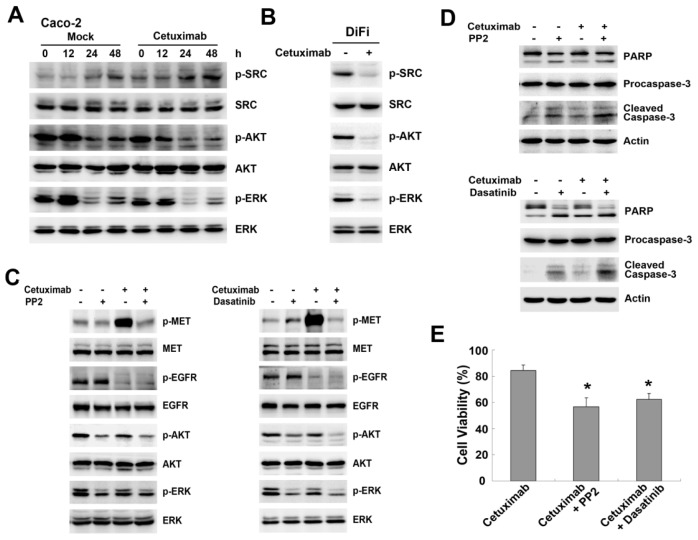
Role of SRC kinase in regulation of delayed MET phosphorylation. (**A**,**B**) Caco-2 cells and DiFi cells were treated with 10 μg/mL cetuximab for the indicated times or 48 h. Expression of SRC, AKT, ERK and phosphorylation levels were analyzed by Western blotting; (**C**,**D**) Caco-2 cells were pretreated with 10 μM PP2 or 0.5 μM Dasatinib for 2 h, and then treated with 10 μg/mL cetuximab for 48 h. Expression of MET, EGFR and downstream targets, and PARP and Caspase-3 were detected by Western blotting; (**E**) Caco-2 cells were pretreated with SRC inhibitors and then subjected to cetuximab for 48 h before analysis with MTT methods. The results from three independent experiments are shown. Student’s *t*-test, * *p* < 0.05 for comparisons between cetuximab-treated and combined treatment.

SRC has been implicated as an intermediate linking EGFR activation to MET phosphorylation [14]. In addition, MET can be activated by SRC in a ligand-independent manner, and SRC activation contributes to EGFR TKI resistance through MET activation [20]. Therefore, we examined the role of SRC in regulating cetuximab-stimulated MET activation. We observed SRC phosphorylation was elevated by cetuximab in a time-dependent manner in Caco-2 cells compared with untreated cells (Figure 4A). Conversely, SRC phosphorylation was inhibited by cetuximab in DiFi cells (Figure 4B). In addition, AKT and ERK phosphorylation was moderately reduced in Caco-2 cells, but substantially attenuated in DiFi cells (Figure 4A,B). These results indicated SRC activation was also involved in resistance to cetuximab, leading us to propose SRC as a mediator of MET activation. Then, we used a selective SRC kinase inhibitor, PP2, and a multi-targeted SFK inhibitor, Dasatinib, to examine the role of SRC in MET activation. Single treatment with PP2 or Dasatinib moderately inhibited AKT and ERK phosphorylation without altering the phosphorylation of MET or EGFR (Figure 4C). Notably, concomitant treatment with SRC inhibitor clearly abolished cetuximab-induced MET activation and led to a further decrease in AKT phosphorylation, suggesting SRC activation was required for MET activation (Figure 4C). Additionally, we found that combination of cetuximab with SRC inhibitor substantially enhanced apoptosis in Caco-2 cells (Figure 4D). Based on these mechanisms, treatment with SRC inhibitor restored the anti-proliferative response to cetuximab in Caco-2 cells (PP2: 56.51% ± 7.04% *vs.* 84.47% ± 4.25%, *p* < 0.05; Dasatinib: 62.33% ± 2.77% *vs.* 87.98% ± 1.69%, *p* < 0.05, Figure 4E). These results indicated that the mutual interaction between MET and SRC was strongly linked in the process of MET activation, thus inhibition of SRC enhanced cetuximab sensitivity through suppressing MET phosphorylation.

### 2.5. Formation of MET/SRC/EGFR Complex Induced by Cetuximab Is Required for MET Activation

**Figure 5.**
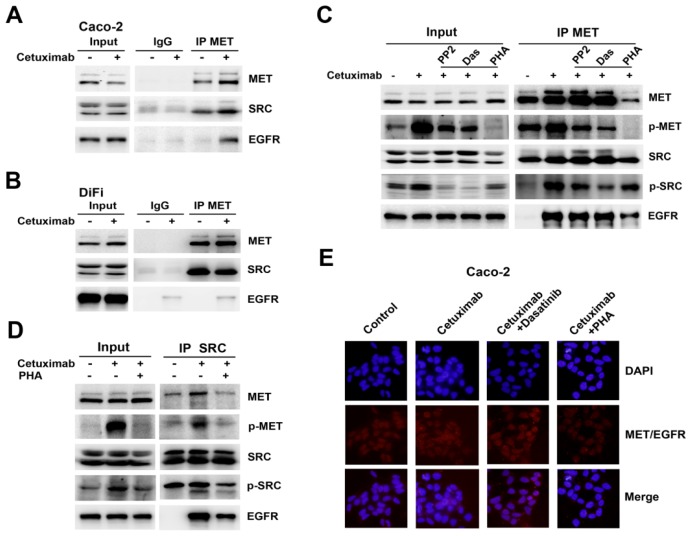
MET/SRC/EGFR complex formation induced by cetuximab is involved in resistance to cetuximab. (**A**,**B**) Whole-cell extracts from Caco-2 cells and DiFi cells treated or untreated with 10 μg/mL cetuximab for 48 h in 2% FBS medium were immunoprecipitated with anti-MET antibody. The immunoprecipitates were probed with MET, SRC and EGFR antibodies. Input represents cell lysates that were not subjected to immunoprecipitation, and control immunoprecipitation was done using IgG mouse; (**C**,**D**) Caco-2 cells were treated with cetuximab or combination with SRC inhibitors (PP2 or Dasatinib) or PHA-665752 for 48 h. The effect on MET/SRC/EGFR complex was analysed by Western blotting using immunoprecipitation with anti-MET or anti-SRC antibodies; (**E**) Heterodimerization of MET and EGFR was detected by Duolink *in situ* PLA (blue, nuclear; red dot, MET-EGFR heterodimer) when exposed to 10 μg/mL cetuximab or combined with Dasatinb or PHA-665752 for 48 h in Caco-2 cells.

EGFR/MET complex associated with SRC were reported in EGFR TKI-resistant breast cancer cells [22]. Therefore, we performed a co-immunoprecipitation assay to elucidate the interaction between MET, SRC and EGFR following cetuximab stimulation. MET co-immunoprecipitation studies revealed a physical interaction between MET and SRC in both Caco-2 and DiFi cells, but EGFR was not associated with MET or SRC in either cell line (Figure 5A,B). The interaction between MET and SRC was enhanced after cetuximab stimulation in Caco-2 cells, whereas binding of MET with SRC was mildly decreased in DiFi cells (Figure 5A,B). Furthermore, we found increased EGFR binding to MET and SRC was induced by cetuximab, resulting in MET/SRC/EGFR complex in Caco-2 cells, whereas no obvious detectable binding was observed in DiFi cells (Figure 5A,B). Next we asked if MET or SRC activation is required for complex formation. Addition of the MET inhibitor PHA-665752 partially affected MET immunoprecipitation, leading to less binding with SRC and EGFR and suppressing MET and SRC phosphorylation (Figure 5C). Similarly, MET inhibition reduced binding of EGFR and MET in the complex, as revealed by immunoprecipitation with anti-SRC antibody (Figure 5D). Unlike the MET inhibitor, the SRC inhibitors could only reduce the phosphorylation of MET and SRC without changing the levels of EGFR and MET in the complex (Figure 5C). The formation of MET/EGFR heterodimers and disruption of complex formation by PHA-665752 were detected by Duolink *in situ* PLA (Figure 5E). The disrupted formation of MET/SRC/EGFR complex by PHA-665752 suggested that MET phosphorylation was an important regulator in cetuximab-induced MET/SRC/EGFR complex.

## 3. Discussion

Cetuximab has been widely used as an effective targeted therapy in CRC for a decade. Although *KRAS* has been identified as an instructive factor for cetuximab response, little is known about the mechanism of cetuximab resistance in wild-type *KRAS* patients. Recently, MET and its ligand HGF were shown to promote resistance to EGFR- and HER2-targeted therapies by activating shared downstream survival and proliferation pathways [10,23]. In this study, we demonstrated that MET activation induced by cetuximab was responsible for cetuximab resistance in colon cancer cells, indicating the benefits of MET inhibition as a novel therapeutic strategy to overcome MET-driven resistance in the clinic.

First, we observed that cetuximab resistance was not associated with basal MET or SRC protein expression or phosphorylation in colon cancer cells. Following treatment with cetuximab at different time points, MET phosphorylation was upregulated in cetuximab-insensitive Caco-2 cells, but not in cetuximab-sensitive DiFi cells. Taking account that the EGFR and MET pathways share similar downstream survival and proliferation pathways, MET activation induced by cetuximab would enable Caco-2 cells to escape the inhibitory effect of cetuximab by activating downstream pathways.

The immediate consequence of cetuximab-induced MET activation was confirmed by inhibition of MET with PHA-665752 and knockdown of MET expression. MET inhibition restored the anti-proliferative and apoptotic effects of cetuximab, revealing that MET activation could compensate for the loss of EGFR signaling. A recent study reported the emergence of MET amplification during anti-EGFR therapy eventually limited the efficacy of further treatment in colon cancer [24]. In the present study, Caco-2 cells, which do not have amplified or mutant MET, exhibited MET activation leading to a functional MET protein capable of activating downstream proliferation and apoptosis pathways. Thus, our results suggested that MET activation induced by cetuximab conferred resistance to cetuximab in non-MET-amplified colon cancer cells, identifying a subset of colon cancer patients who develop cetuximab resistance in the absence of MET amplification.

SRC activity serves as an essential mediator for cross talk among tyrosine kinase receptors including EGFR and MET [14,20,25], and SRC family kinases have become an attractive and promising target for CRC therapy [17]. In this study, we observed SRC phosphorylation was coupled with MET activation triggered by cetuximab in Caco-2 cells, denoting the tight interaction of MET with SRC in cetuximab resistance. Disruption of SRC activity with inhibitors resensitized Caco-2 cells to cetuximab, indicating that SRC activation contributed to cetuximab resistance, consistent with previous findings [17]. Additionally, a feedback loop between MET and SRC was confirmed in the process of cetuximab-induced MET activation. Inhibition of MET decreased SRC phosphorylation, and conversely inhibition of SRC suppressed MET phosphorylation. Moreover, previous studies have demonstrated that cooperation of MET with SRC mediates resistance to EGFR TKIs [15]. Taken together with these reports in the literature, our data indicated that SRC activation is more dependent on MET signaling than on EGFR signaling in EGFR inhibitor-resistant cells, and further support the importance of MET/SRC interaction as a common molecular mechanism to evade anti-EGFR treatment. These results provided a mechanistic foundation for alternative SRC-directed strategies or combination treatment targeting both MET and SRC in cetuximab-resistant patients.

**Figure 6.**
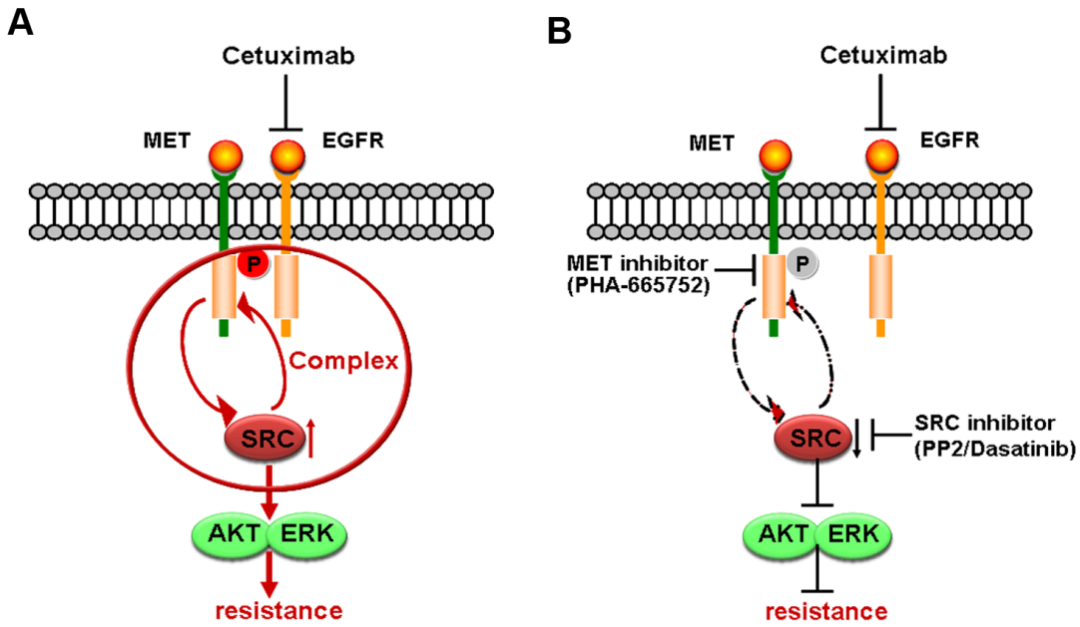
Schematic representation of the proposed model involved in cetuximab-induced MET activation. (**A**) Application of cetuximab promotes MET phosphorylation coupled with SRC activation in Caco-2 cells. The positive feedback between MET and SRC and formation of MET/SRC/EGFR complex heighten cetuximab resistance; (**B**) Inhibition phosphorylation of MET and SRC by inhibitors disrupts the interaction of MET with SRC and formation of MET/SRC/EGFR complex, ultimately reversing cetuximab resistance.

It is reported that formation of complex or heterodimers between different receptors can stimulate intracellular signaling components in a distinct pattern that confers resistance to targeted agents [12,19]. In cells developing acquired resistance to cetuximab, activation of EGFR along with other transmembrane receptors (HER2, HER3 and MET) leads to transactivation [19]. Our co-immunoprecipitation results confirmed that cetuximab induced formation of MET/SRC/EGFR complex in Caco-2 cells, but not in DiFi cells. Moreover, disruption of the association of MET/SRC/EGFR by MET or SRC inhibitors reduced cetuximab-induced phosphorylation of MET and SRC, and MET inhibition reduced complex formation (Figure 6A,B). Therefore, it was plausible that cetuximab-mediated MET/SRC/EGFR complex played a crucial role in promoting activation of MET and its downstream pathways. Similarly, in acquired cetuximab-resistant colon cancer cells, overexpression of TGF-α induced the EGFR-MET interaction with subsequent MET phosphorylation, which contributed to cetuximab resistance [26]. Given that kinase-inactive EGFR is able to interact with and stabilize several cancer-relevant proteins [14], we speculated the redundant EGFR not bound to cetuximab in Caco-2 cells might further interact with MET in spite of kinase-inactivation by cetuximab inhibition, resulting in enhancement of the MET pathway and close associations between MET and SRC.

## 4. Experimental Section

### 4.1. Cell Culture and Reagents

Colon cancer SW480, DLD-1, HCT-116, HT-29, RKO and Caco-2 cells were obtained from the Type Culture Collection of the Chinese Academy of Sciences (Shanghai, China). DiFi cells were purchased from Shanghai Yan Cheng biological technology Co., Ltd. (Shanghai, China). SW480 cells were grown in Leibovitz’s L-15 medium (Gibco, Gaithersburg, MD, USA), DiFi cells were grown in McCoy’s 5A medium (Gibco, Gaithersburg, MD, USA), and all the other cells were cultured in Roswell Park Memorial Institute (RPMI) 1640 medium (Gibco, Gaithersburg, MD, USA). Media were supplemented with 10% fetal bovine serum (FBS). Cetuximab was obtained from Merck KgaA (Darmstadt, Germany). PHA-665752 and PP2 were purchased from Sigma-Aldrich (St. Louis, MO, USA). Dasatinib was purchased from Selleck chemicals (Houston, TX, USA). Antibodies to MET, phospho-MET (Tyr1234/1235), EGFR, phospho-EGFR (Tyr1068), SRC, phospho-SRC (Y416), poly (ADP-ribose) polymerase (PARP), AKT, phospho-AKT (Ser473), phospho-ERK1/ERK2 (Thr202/Tyr204), Caspase-3 were obtained from Cell Signaling Technology (Beverly, MA, USA). Anti-Actin, anti-ERK, secondary goat anti-rabbit and goat anti-mouse antibodies were purchased from Santa Cruz Biotechnology (Santa Cruz, CA, USA).

### 4.2. Cell Viability Assay

The effect of cell proliferation was measured using a 3-(4,5-dimethyl thiazol-2-yl)-2,5-diphenyl tetrazolium bromide (MTT) assay. Briefly, the cells were seeded in triplicate at 4000–8000 cells/well in 96-well plates and incubated at 37 °C for 24 h in 10% FBS medium. After pre-incubation with 2% FBS medium overnight, the cells were treated with indicated doses of cetuximab singly or in combination with PHA-665752, PP2 or Dasatinib for 48 or 72 h. Then, 25 μL of MTT solution (5 mg/mL) was added to each well and the cells were incubated for another 4 h at 37 °C. After the removal of the culture medium, the cells were lysed in 200 μL of dimethylsulphoxide (DMSO) and the optical density (OD) was measured at 570 nm with a microplate reader (Model 550, Bio-Rad Laboratories, Hercules, CA, USA).

### 4.3. Western Blotting

The cells were washed twice with ice-cold phosphate-buffered saline (PBS), lysed in lysis buffer (1% Triton X-100, 50 mM Tris-HCl pH 7.4, 150 mM NaCl, 10 mM EDTA, 100 mM NaF, 1 mM Na_3_VO_4_, 1 mM PMSF, 2 μg/mL aprotinin) and quantified with the Lowry method. Cell lysate proteins were subjected to SDS-PAGE electrophoresis then transferred to a nitrocellulose membrane (Immoblin-P, Millipore, Bedford, MA, USA). After blocking with 5% skim milk in TBST buffer (10 mM Tris-HCl pH 7.4, 150 mM NaCl, 0.1% Tween 20) at room temperature for 2 h, the blots were incubated with indicated antibodies overnight at 4 °C. After washing three times with TBST buffer, the membrane was incubated with secondary antibodies for 30 min at room temperature followed by TBST buffer washing. Finally, the protein bands were detected with enhanced chemiluminescence reagent (SuperSignal Western Pico Chemiluminescent Substrate; Pierce, Rockford, IL, USA) and visualized with the Electrophoresis Gel Imaging Analysis System (DNR Bio-Imaging Systems, Jerusalem, Israel).

### 4.4. Fluorescence Microscopy

Cells were seeded at 120,000 cells/well and treated with 10 μg/mL cetuximab for 48 h in 2% FBS medium in Lab-Tek chamber slides (Nunc S/A, Polylabo, Strasbourg, France). Cells were then fixed with 3.3% para-formaldehyde for 20 min, permeabilized with PBS buffer containing 0.2% Triton X-100 for 3 min and blocked with 5% bovine serum albumin (BSA). The slides were incubated with anti-phospho-MET (Tyr1234/1235) antibody for 2 h and then incubated with fluorescein isothiocyanate (FITC)-conjugated goat anti-rabbit IgG for 45 min. The nucleus was counter-stained with DAPI (NACALAI TESQUE. INL) for 5 min, then observed under a fluorescence microscope (Olympus, ELNA, Tokyo, Japan).

### 4.5. Clonogenic Assay

Cells were seeded at 300 cells/well in 12-well plates and treated with either 10 μg/mL cetuximab or 0.4 μM PHA-665752, or co-treatment with cetuximab and PHA-665752 after plating for 24 h in 10% FBS medium. Cells were allowed to grow for additional 14 days. Followed by staining with Wright Giemsa, the number of colonies was counted.

### 4.6. Small Interfering RNA Transfections

Two different pairs of MET siRNA from Shanghai Gemma pharmaceutical technology Co., Ltd. (Shanghai, China) were used: 5′-GCCUGAAUGAUGACAUUCU-3′ and 5′-GUCCCGAGAAUGG UCAUAA-3′. The siRNAs were transfected with Lipofectamine 2000 (Invitrogen, Carlsbad, CA, USA) per the manufacturer’s instructions.

### 4.7. Co-Immunoprecipitation

Co-immunoprecipitations were performed using 200 μg of cell lysates with 5 μL of mouse anti-MET or anti-SRC antibody or control IgG mixed with Protein G Agarose beads (GE Healthcare Bio-Sciences AB, Pittsburgh, PA, USA) in a final volume of 500 μL of lysis buffer with gentle rocking overnight at 4 °C. The beads collected were spun down for 1 min at 14,000/rpm and washed four times with lysis buffer. Then 40 μL Sampling buffer was added, boiled at 96 °C for 5 min and subjected to Western blotting.

### 4.8. In Situ Proximity Ligation Assay

*In situ* proximity ligation assay (PLA) was done to detect MET-EGFR heterodimer. Cells were seeded and treated with cetuximab or combined with Dasatinib or PHA-665752 for 48 h in 2% FBS medium in Lab-Tek (Waltham, MA, USA) chamber slides. We used Duolink *in situ* PLA (Olink Bioscience, Uppsala, Sweden) according to the manufacturer’s instructions. Anti-MET Ab and anti-EGFR Ab were used as primary antibodies. The nucleus was stained with DAPI for 5 min. The specimens were observed using a fluorescence microscope (Olympus, ELNA, Tokyo, Japan).

### 4.9. Statistical Analysis

All values are expressed as the mean ± standard deviation (S.D.). The differences of the results between two groups were evaluated by Student’s *t*-test. SPSS 16.0 computer software (SPSS Inc., Chicago, IL, USA) was used for statistical analysis. *p*-value of 0.05 or less was considered statistically significant.

## 5. Conclusions

In summary, we showed that MET activation induced by cetuximab is a novel resistance mechanism to cetuximab in colon cancer cells. Additionally, the feedback between MET and SRC and the formation of MET/SRC/EGFR complex are both essential mechanisms involved in cetuximab-induced MET activation. These results implicate the critical role of the MET pathway in mediating resistance to cetuximab and provide a theoretical basis for further investigation of combinatorial strategies with MET or SRC inhibitors in patients exhibiting cetuximab-resistance. Future studies will focus on the therapeutic significance of these findings, which will be highlighted by an expanded preclinical experiment to assess MET phosphorylation by immunohistochemistry in xenografted tumors and clinical samples from cetuximab-resistant cases.

## Supplementary Information
