# Supplementary material for: Cetuximab-Induced MET Activation Acts as a Novel Resistance Mechanism in Colon Cancer Cells
Source: Int J Mol Sci. 2014 Apr 4;15(4):5838–51. doi: 10.3390/ijms15045838 (PMC4013599; doi:10.3390/ijms15045838)
Supplement: Supplementary file 1 [file ijms-15-05838-s001.pdf]

Supplementary Information

Table S1. Summary of cetuximab sensitivity and genotypical data of seven kinds cell lines used in experiments.

Cell line	Cell viability (%) at 10 μg/mL 72 h	Cetuximab sensitive	KRAS mutation	BRAF mutation	PIK3CA mutation	PTEN deficiency
SW480	98.25 ± 0.33	No	Yes	WT	WT	No
HCT-116	102.54 ± 2.38	No	Yes	WT	Yes	No
DLD-1	85.91 ± 2.38	No	Yes	WT	Yes	No
HT-29	89.16 ± 1.74	No	WT	Yes	WT	No
RKO	85.18 ± 2.24	No	WT	Yes	Yes	No
Caco-2	77.06 ± 3.78	No	WT	WT	WT	No
DiFi	50.78 ± 6.81	Yes	WT	WT	WT	No

© 2014 by the authors; licensee MDPI, Basel, Switzerland. This article is an open access article distributed under the terms and conditions of the Creative Commons Attribution license (http://creativecommons.org/licenses/by/3.0/).
